# Manual head rotation synchronised to a metronome is a feasible and valid method for assessing visually enhanced vestibulo-ocular reflexes and vestibulo-ocular reflex suppression

**DOI:** 10.3389/fneur.2026.1706773

**Published:** 2026-04-22

**Authors:** Thomas Ming Hong Chang, Richard H. Roxburgh, Rachael L. Taylor

**Affiliations:** 1 Centre for Brain Research Neurogenetics Research Clinic, University of Auckland, Auckland, New Zealand; 2 Auckland City Hospital, Auckland, New Zealand; 3 Department of Neurology, Auckland City Hospital, Auckland, New Zealand; 4 Department of Audiology, University of Auckland, Auckland, New Zealand

**Keywords:** metronome, vestibulo-ocular reflex, vestibulo-ocular reflex suppression (VOR-S), video-oculography, visually enhanced vestibulo-ocular reflex (VVOR)

## Abstract

**Introduction:**

The visually enhanced vestibulo-ocular reflex (VVOR) and vestibulo-ocular reflex suppression (VOR-S) are critical in evaluating vestibular and cerebellar contributions to oculomotor control. This study aimed to investigate the feasibility and reliability of metronome-synchronised, manual head rotation video-oculography for evaluating VVOR and VOR-S in healthy participants. A secondary aim was to determine the effects of head rotation frequency and direction, and participant’s age, on VVOR and VOR-S gain and saccades.

**Methods:**

Twenty-four healthy individuals aged 20–79 years underwent VVOR and VOR-S assessments performed over a range of frequencies from 0.25 to 1.25 Hz, using a portable ICS Impulse video-oculography device.

**Results:**

Good control over the frequency and velocity of head rotation was achieved using the metronome. Correlations with the intended frequency (Spearman’s rho) ranged from 0.876 to 0.884. The VVOR gains were all close to 1.0 and were unaffected by age or the frequency of head rotation. Saccade rates for VVOR were low. The VOR-S gains and refixation saccades increased with increasing frequency and there was a small but significant effect of age. Slightly higher gains were recorded for head turns to the right for both the VVOR and VOR-S.

**Discussion:**

The findings confirm that use of a metronome can effectively control the frequency and to an extent, the velocity of head rotation, offering a cost-effective alternative to traditional rotational chair testing of the VVOR and VOR-S. The results provide a basis for future research and clinical application as a reference for identifying cerebellar and combined cerebellar and vestibular dysfunction.

## 1 Introduction

Different classes of voluntary and reflexive eye movements function during daily activities to ensure that the visual world remains centred on the fovea where acuity is best. These eye movements are critically dependent on specific brain regions, and some involve sensory input from the vestibular system. Consequently, the examination and recording of eye movements is a mainstay in the assessment of neurological and vestibular disorders.

The vestibulo-ocular reflex (VOR) stabilises vision during head movement by generating approximately equal compensatory eye movements in the direction opposite the head movement. The angular (or rotational) VOR relies on input from the semicircular canals of the inner ear, which detect angular head acceleration and relay information via the vestibular nucleus to the oculomotor nuclei. The visually enhanced vestibulo-ocular reflex (VVOR) combines the VOR with visually evoked, cerebellar-mediated eye movements (1). Retinal image slip during low-velocity (<100°/s) head perturbations triggers tracking eye movements (smooth pursuit and optokinetic reflexes), which reinforce fixation of the eyes to ensure stationary images remain centred on the fovea (1–3).

In contrast to its additive role in the VVOR, the cerebellum contributes to suppression of the VOR (VOR-S) when it is detrimental to vision. For example, moving one’s head to track a ball as it is kicked across a sports field elicits a VOR, which will unproductively tend to move the eyes in the opposite direction, away from the ball. Unopposed, this would result in loss of foveal centring. To prevent this from occurring, small image drifts across the retina lead to increased activation of cerebellar Purkinje cells to modify the output from the vestibular nucleus, reducing the drive to the extra-ocular muscles (4). Recording the VOR, VVOR and VOR-S provides complementary diagnostic information. VOR abnormalities reflect vestibular dysfunction, VOR-S abnormalities reflect disorders of the cerebellum, especially those involving the vestibulo-cerebellum and dorsal vermis (4), and VVOR abnormalities to low-velocity head movement reflect combined vestibular and cerebellar function impairment (1, 2, 5).

Eye movements can be assessed by visual examination at the bedside. While convenient, this approach is not quantifiable and so cannot be used for monitoring changes over the course of a disease. Technological advances in video-oculography have facilitated development of portable devices, capable of providing a repeatable and reliable measure of the VOR to high-velocity manual head rotation – widely referred to as the video head impulse test (vHIT) (6). This method of assessment is easily implemented in the small office practice or outpatient clinic using established methods and published normative data. In contrast, the best validated method for assessing the VVOR and VOR-S involves use of a motorised swinging chair, which is available only in specialised neuro-otology clinics and research centres.

The same video-oculography technology used for vHIT can also be used to assess the VVOR and VOR-S to low-velocity manual head rotation (7, 8). Publications now include examples of abnormal VVOR recordings in patients with combined vestibular-cerebellar disorders, which typically show multiple refixation saccades and eye velocity traces that appear reduced compared with the corresponding head velocity (9, 10). However, a limitation of these portable devices is they do not provide any means of controlling the frequency of manual head rotation, nor any quantitative VVOR or VOR-S measurements. Instead, the clinician attempts to approximate a low-velocity, sinusoidal head movement and interpret the recordings based on their subjective impression of the eye and head velocity traces. This qualitative interpretation has precluded a good understanding of the feasibility of the manual rotation method, the effect of different procedural variables and associated technical pitfalls.

The development of open access analysis software by Rey Martinez et al. (7) provides a useful complement to portable video oculography, providing a quantitative means of studying VVOR and VOR-S outcomes. Using this approach, Soriano-Reixach et al. (11) investigated the effects of demographic variables in 19 healthy participants, identifying head rotation frequency as a significant determinant of VOR-S gains. However, neither the frequency nor the amplitude of head movement was strictly controlled, resulting in oscillation frequencies ranging from 0.8 to 1.8 Hz. Thus, the feasibility and reliability of manual head-rotation at lower frequencies, which are likely to be important for ensuring the specificity of VVOR and VOR-S abnormalities, remain uncertain. The few studies investigating objective VVOR and VOR-S outcomes to manual head rotation in clinical populations, mainly in peripheral vestibular disorders, have similarly shown variable control over head frequency and velocity (8, 12). A recent study has suggested that control over rotation frequency could be achieved through use of a metronome (13). However, the corresponding head velocities, and degree to which these could also be controlled, was not investigated.

This study extends previous research by refining the method of manual head rotation for improved precision and objectivity of VVOR and VOR-S testing using portable video oculography. Firstly, we evaluate the feasibility and reliability of manual head rotation in healthy subjects, synchronised to a metronome, at a range of head velocities over which vestibular and cerebellar function contribute to gaze stabilisation. It was hypothesised that use of a metronome and visual targets would help to ensure consistent pacing and movement amplitude, leading to more consistent head velocities compared with previous studies. A second objective was to quantify the effects of head rotation frequency, direction and age on the gain and rates of refixation saccades, and to establish reference values for these measures for future comparison with clinical populations.

## 2 Methods

### 2.1 Participants

Twenty-four healthy adults (12 males and 12 females; aged 20–79) were recruited for the study. All participants underwent a detailed medical history review and examination to exclude any neurological, oculomotor or vestibular conditions, and video head impulse testing (vHIT) confirmed normal horizontal semicircular canal function (gain > 0.8). Informed consent was obtained from all participants, and ethical approval was provided by the Auckland Health Research Ethics Committee (AHREC) on 18/02/2020 (Reference AH1110).

### 2.2 Video-oculography and data collection

The ICS Impulse (Natus Sensory) video-oculography device and Otosuite software (version 4.1) was used to record head and eye movements. The equipment consists of a lightweight (60 g), tight-fitting pair of goggles worn by the participant which connects via USB port to a laptop. Within the goggles is a 3D inertial sensor, and within the right eye frame is a small video camera and a half-silvered mirror which reflects the image of the eye onto the camera.

The software processes head and eye velocity data to generate graphical traces that can be viewed in real-time and after testing. Version 4.1 of the Otosuite software automatically calculates the gain (the ratio of eye to head velocity) of the VOR for vHIT, but not for the VVOR or VOR-S. Therefore, raw VVOR and VOR-S data were exported as a comma separated values (CSV) file format and analysed offline in MATLAB, using an analysis program developed by Rey-Martinez et al. (7). The program was downloaded in its entirety as an open-source MATLAB script from the GitHub web repository. Gain values were calculated using the same “Area Under the Curve” method that is used for calculation of vHIT VOR gains within the Otosuite software.

### 2.3 Participant positioning and test configuration

**Figure 1:**
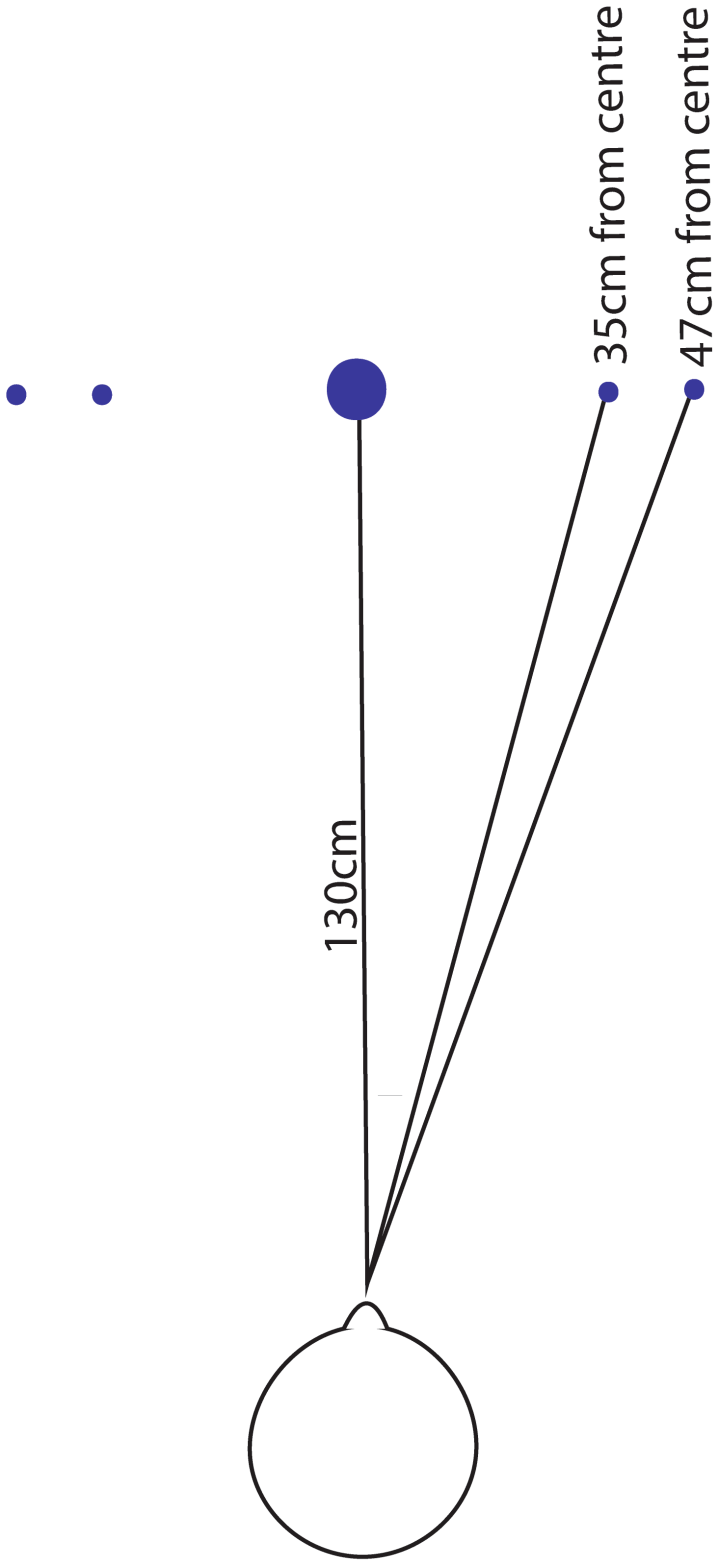
Wall-marker set-up for VVOR and VOR-S. Schematic of the central visual fixation target and the smaller visual guides as measured from the nasion. The examiner attempted to control the angle of head movement so that each left and right turning point fell between each set of visual guides.

Participants were seated comfortably in a straight-backed chair positioned 1.3 m from an undecorated wall (Figure 1). This distance was chosen such that head rotation could subtend an angle of 15–20 degrees on each side without the pupil of the eye running outside the region of interest on the Otosuite software. Assessment of the VVOR involved the use of a centrally positioned visual target that was fixed to the wall at eye level; we used the generic blue logo supplied by Natus Sensory. A moving laser-dot projected onto the wall from the video goggles served as a head-referenced target for VOR-S. To assist the examiner in controlling the amplitude of the head movement, two small, light-coloured markers (Blu Tack - Bostik, UK) were positioned at 35 cm and 47 cm either side of the wall-fixed target [corresponding to an angle of 15 and 20 degrees from the participants nose bridge (see Figure 1)]. Since the axis of head rotation is approximately 10 cm posterior to the nasion, the angle for a head turn falling in between the two markers was predicted to range from 14 to 18.5 degrees either side of the midline. From these angles, we were able to calculate the approximate peak-head velocity range corresponding to different head rotation frequencies (Supplementary Table 1) using the Equation 1:


$$V_{\text{peak}}=2\mathrm{\pi fA}$$

### 2.4 Procedure

First, the recording goggles were securely fastened to the participant’s head. The examiner then stood behind the participant with both hands on top of the participant’s head which was manually rotated at frequencies of 0.25, 0.50, 0.75, 1.00, and 1.25 Hz, synchronised to a metronome (Pro Metronome – Tempo & Tuner) downloaded from the Google App store onto an iPhone. The metronome was only audible to the examiner via an earphone placed in one ear. The order of test frequencies was randomised for each participant.

During head rotation, the participant was instructed to fixate on the visual target, which was either the stationary, earth-fixed mark for the VVOR, or the head-fixed laser-dot for the VOR-S. During the VOR-S the projected laser dot moved in unison with the head, allowing the examiner to control the amplitude of oscillation by landing the laser between the pre-measured Blu Tack markers on the wall at each turning point. Therefore, VOR-S was always performed first. During the VVOR, when the laser was turned off, the examiner needed to estimate the amplitude by attempting to align the nose between the centre of the markers during each head turn. Encouragement to concentrate and fixate on the target was given throughout since this reinforcement has been shown to produce a small but significant improvement in VOR-S (i.e., a slightly lower gain) (11).

### 2.5 Raw data analysis

Individual CSV files exported from Otosuite were imported into MATLAB. First, the analysis program was run on the entire time trace and results were examined alongside the saved video recording to check for any disruptive artefacts. The same artefacts that can interfere with vHIT testing, such as loss of pupil tracking and excessive blinking, can also affect VVOR and VOR-S testing. Next, the analysis command was repeated but on a selected timeframe that was free of eye closure or other types of artefacts. Criteria for time frame selection included capture of a minimum of five cycles, exclusion of artefacts, and a good sinusoidal head movement profile. Where possible, an intermediate time interval (after the head has started moving and before it has stopped) was selected, as this has been shown to be more reliable (11). The information extracted from the analysis included the gain, rates of refixation saccades (per second), the frequency of head rotation, and the average peak-head velocity.

### 2.6 Qualitative and quantitative statistical analysis

Raw head velocity traces were visually inspected for any deviation from a smooth sinusoidal profile. Next, the strength of the relationship between the intended and the actual frequency of head rotation, as well as between the intended frequency and the actual head velocity, was explored using Spearman’s correlation coefficient.

The VVOR and VOR-S gains were compared in SPSS using a General Linear Mixed Model (GLMM), assuming a diagonal covariance structure (based on comparison of likelihood ratios). The fixed effects included in the model were the direction (left or right) and frequency (five levels) of head rotation. Age was included as a covariate, and participant as a random effect. *Post hoc* analyses for significant main effects were performed using paired comparisons of the estimated marginal means, using the 0.50 Hz frequency as the reference category. We chose 0.50 Hz because it has been recommended for VVOR testing in patients with the clinical syndrome of cerebellar ataxia with neuropathy and bilateral vestibular areflexia (CANVAS) (2). The *p*-values were adjusted using Bonferroni corrections to control for multiple comparisons.

## 3 Results

### 3.1 Qualitative analysis

**Figure 2:**
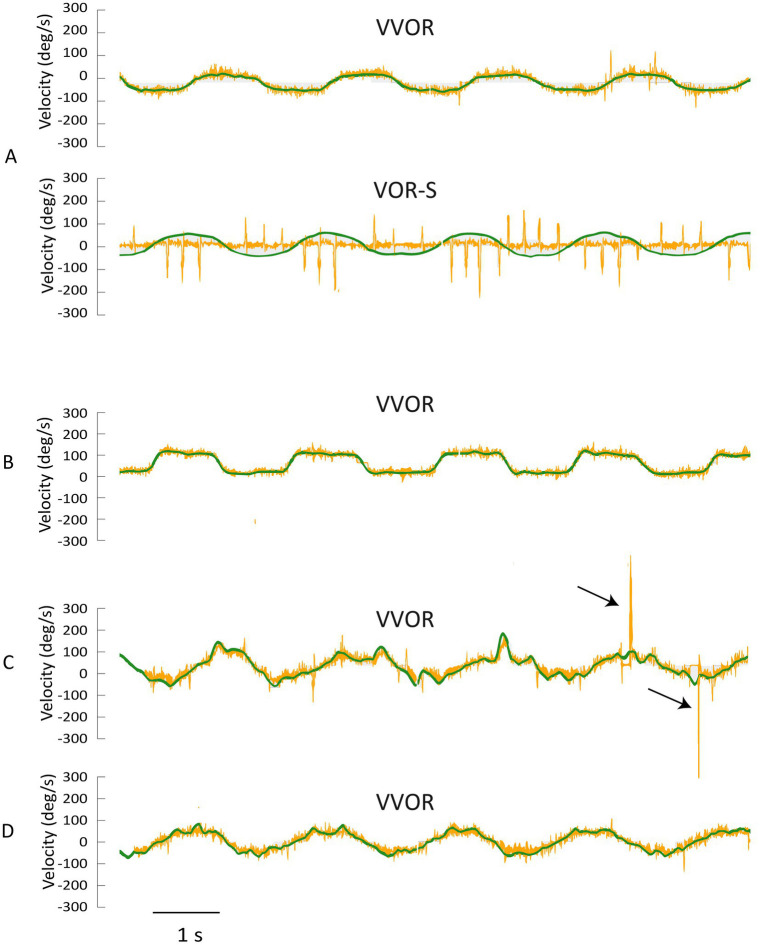
Representative head velocity profiles. **(A)** Shows representative sinusoidal VVOR and VOR-S head (green) and eye (yellow) velocity profiles recorded in most participants. The velocity profile in **(B)** shows a square rather than sinusoidal VVOR profile. **(C)** Demonstrates a jerky VVOR head movement, which improved slightly in after re-instructing the participant to relax **(D)**.

Representative velocity traces typical of 22 of the 24 participants VVOR and VOR-S are shown in Figure 2A (see Supplementary Videos S1, S2). In both examples, the traces have a consistent sinusoidal profile that approximates the intended head rotation frequency. Increased neck resistance was encountered in 6/24 participants and for two of these participants it affected their head velocity profile (Figures 2B,C; Supplementary Videos S3, S4). For the example in (Figure 2C), the profile improved somewhat after the participant was re-instructed to try and relax (Figure 2D; Supplementary Video S5). Shifting the examiners hand placement from the top of the head to the jaw also improved the head velocity profile in some cases.

### 3.2 Reliability analyses

**Figure 3:**
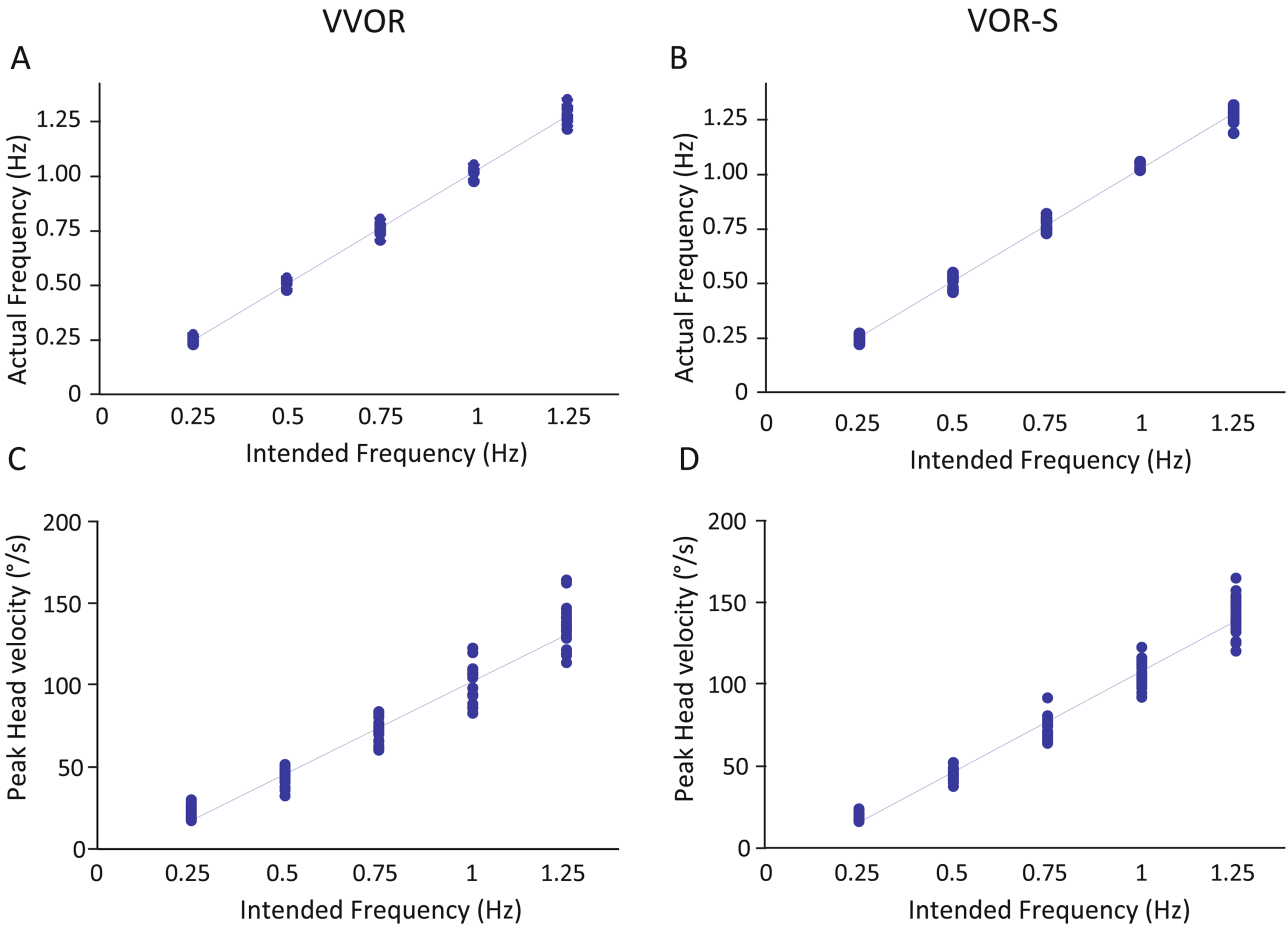
Relationship between intended and actual head rotation frequency and peak velocity. The correlation between intended and actual frequency of head rotation are shown for the VVOR **(A)** and VOR-S **(B)**. **(C,D)** show the corresponding correlation between the intended frequency and the recorded velocity of head rotation.

The results of the actual frequencies and peak velocities of head motion relative to the intended frequencies are presented in Figure 3 and Supplementary Table 1. There was a strong positive correlation between the actual and intended head frequency (Spearman’s rho = 0.884 for both VOR-S and VVOR, *p* < 0.01), confirming excellent control over the frequency of head oscillation. The relationship between head frequency and peak head velocity was slightly more variable, particularly at higher frequencies of head rotation. However, strong correlations of 0.882 and 0.876 (*p* < 0.01) were still observed for VOR-S and VVOR, respectively.

### 3.3 Effects of head rotation frequency and direction

**Figure 4:**
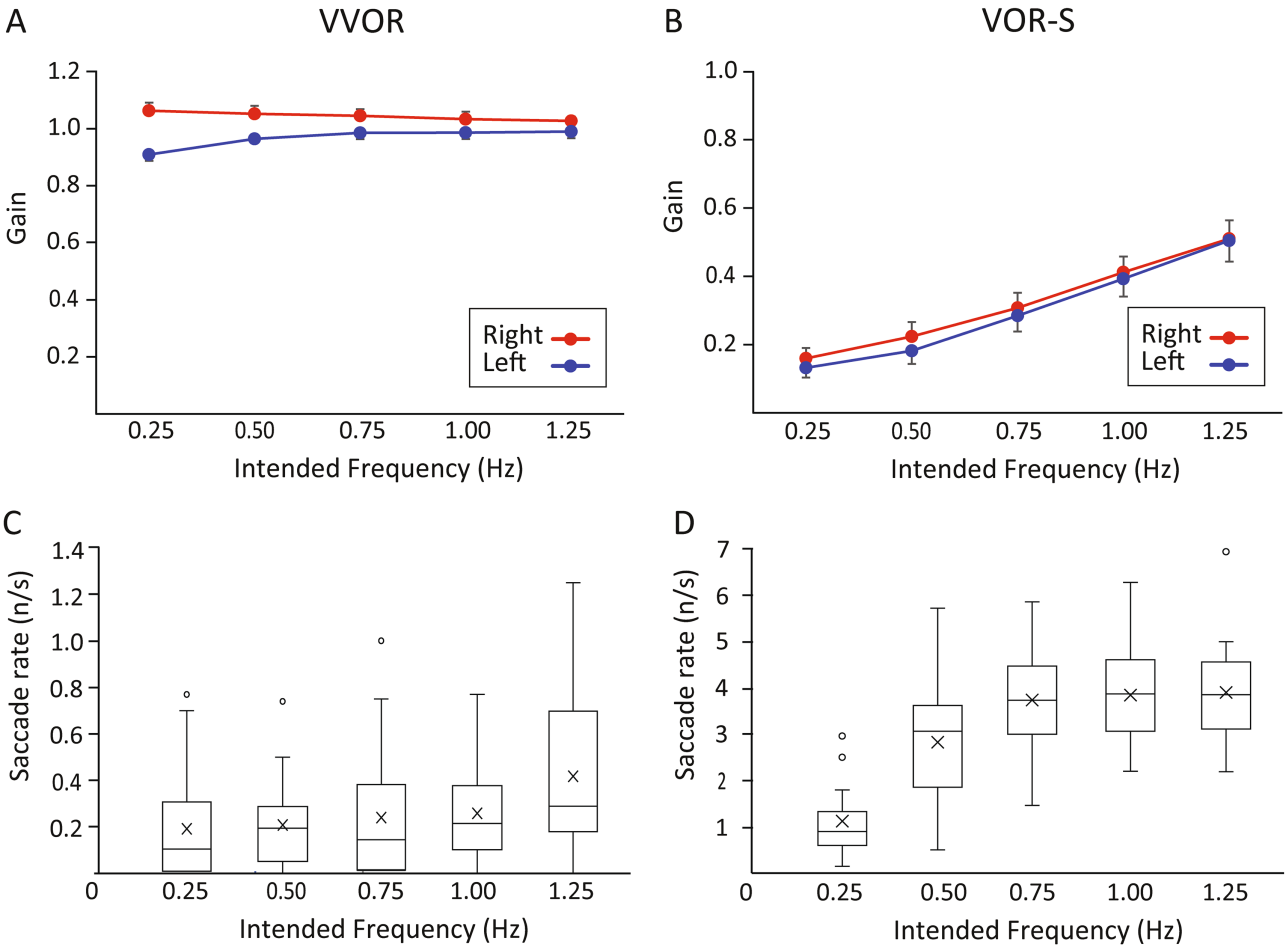
Effect of head rotation frequency and direction on VVOR and VOR-S gain and refixation saccades. Estimated marginal means (EMM) and error bars (2SE) for gains produced by head rotation to the right (red) and left (blue) are shown as a function of the intended frequency for VVOR **(A)** and VVOR-S **(B)**. **(C,D)** Show the corresponding box and whisker plots comparing the rate of saccades across the different frequencies. Mean values for each frequency are represented by a cross; outliers are represented by open circles.

The results of VVOR and VOR-S gain and saccade analyses are summarised in Figure 4 and Supplementary Tables 2, 3.

#### 3.3.1 VVOR

The VVOR gain was close to 1.0 at all rotation frequencies, meaning that eye velocity approximately matched head velocity, with no evidence of any difference across frequency [*F*_(4, 79.484)_ = 1.758 *p* = 0.146]. However, there was a significant interaction effect between the direction and frequency of head rotation [F_(4, 79.484)_ = 7.320 *p* < 0.001]. As shown in Figure 4, VVOR gains were on average higher for head rotations to the right (mean 1.044, 95% CI 1.032–1.055) than to the left (mean 0.967, 95% CI 0.957–0.977), consistent with a significant main effect of direction [*F*_(4, 160.470)_ = 96.752 *p* < 0.001]. However, the magnitude of the difference between the two directions decreased as frequency increased. At 0.25 Hz, gains were 0.154 higher for head movement to the right (*p* < 0.001), whereas at 1.25 Hz the difference decreased to 0.037 (*p* = 0.044). There was no evidence that gain changed with age [*F*_(1, 221.469)_ = 0.001 *p* = 0.981].

Median saccade rates for the VVOR were low overall, ranging from 0.11 to 0.29 saccades per second (Figure 4). Analysis of the effect of frequency on rates of saccades revealed a significant difference among the five levels (chi-square = 18.113, df = 4, *p* = 0.01), with a trend for slightly higher rates at higher frequencies of head rotation. For example, the median rate was 0.29/s at 1.25 Hz compared with 0.11/s at 0.25 Hz. However, Wilcoxon signed-rank tests between 0.50 Hz (the predesignated reference category) and each of the other frequencies revealed no significant pairwise differences.

#### 3.3.2 VOR-S

The VOR-S gains increased with higher frequencies of head rotation [*F*_(4, 86.049)_ = 90.914, *p* < 0.001]. *Post-hoc* pairwise comparisons against the 0.50 Hz reference category (mean gain = 0.20) confirmed significantly lower gains at 0.25 Hz (*p* = 0.006), whereas gains recorded at 0.75, 1.00 and 1.25 Hz were all significantly higher (*p* < 0.001).

The effect of head rotation direction was also significant [*F*_(1, 186.022)_ = 4.349, *p* = 0.038], where VOR-S gains were overall slightly higher for head rotations to the right (mean 0.324, 95% CI 0.305–0.342) than to the left (mean 0.297, 95% CI 0.278–0.316) (Figure 4). Age as a covariate exhibited a significant effect on gain (*p* = 0.019) where for every 10-year increase in age, there was a small increase in gain of 0.008.

Median rates of saccades (range: 0.90 to 3.86/s) shown in Figure 3 increased with increasing head rotation frequency until 0.75 Hz when they begin to plateau. Friedman test confirmed a significant difference among the five frequencies (chi-square = 57.467, df = 4, *p* < 0.001). Comparisons against the 0.50 Hz reference category confirmed a significantly lower rate of saccades at 0.25 Hz (adjusted *p* < 0.001), whereas rates recorded at 0.75, 1.00 and 1.25 Hz were all significantly higher (adjusted *p* = 0.004). There was no evidence of a relationship between the rate of saccades and age (rho = 0.029; *p* = 0.754).

## 4 Discussion

This study demonstrates that a simple manual head rotation protocol, synchronised with a metronome enables the generation of sinusoidal head movements close to intended frequencies and within a discrete range of velocities. Compared with a previous study which employed neither frequency nor amplitude control (7), addition of the metronome led to substantially increased precision and reduced variability in both the recorded frequencies and velocities. For example, at the intended frequency of 1 Hz, the standard deviations for the recorded frequencies and velocities of VVOR head movements were 0.01 Hz and 13.1°/s respectively, whereas in the previous study of healthy controls they were 0.32 Hz and 36.2°/s. Our study also provides normative data for VVOR and VOR-S gain and rates of refixation saccades for low head velocities < 100 °/s, critical for the assessment of cerebellar and combined vestibular-cerebellar disorders.

### 4.1 Feasibility and reliability of synchronised manual head rotation

Compared with rotational chair testing, manual head rotation and video-oculography offer several practical advantages including portability, cost effectiveness and feasibility for use in a small office practice or outpatient clinic. Thus, there is the potential for more widespread quantitative VVOR and VOR-S assessment. However, despite our positive results confirming good control over the frequency and velocity of head rotation, there are some limitations to our method.

It was observed that with increasing head rotation frequency, the variability of head rotation velocity increased. Control over head rotation velocity, not just the frequency, is critical to ensure that VVOR abnormalities remain specific to combined vestibular and cerebellar dysfunction (1). This is due to the relatively narrow velocity range over which vestibular and smooth pursuit eye movements overlap, typically below 100°/s (3). While optokinetic responses can also contribute, these reflexes are best evoked by full-field visual scene motion (1). Therefore, in the context of VVOR testing, smooth pursuit is likely to be the dominant non-vestibular contributor to visual fixation. Variability in head velocity was less of an issue for the VOR-S, probably because the rotation amplitude is controlled by landing a laser dot within target ranges, whereas the VVOR provides no pointer to aim with.

Neck stiffness was encountered in six (25%) of the 24 participants and for two of these participants, this resulted in head velocity profiles that were neither smooth nor sinusoidal. While re-instruction led to some improvement, these physical constraints can be challenging to overcome using manual rotation methods and it is possible that neck stiffness could be exacerbated in patient populations (5). The issue was highlighted by Halmagyi et al. (5) in a recent letter to the editor outlining their experience with VVOR and VOR-S testing in patients with CANVAS syndrome and gentamicin vestibulotoxicity. The difficulty they encountered in producing a consistent head oscillation at a defined low-frequency and velocity led them to abandon manual rotation in favour of rotational chair testing. These observations highlight the potential value of our approach, which prioritises controlled low-velocity testing, showing that this was achievable in most healthy participants. Whether the method could similarly benefit patient populations in which altered muscle tone, head movement restriction and other medical comorbidities are more common, requires further investigation.

Finally, manual head rotation is inherently examiner dependent. Data collection and analysis in our study was undertaken primarily by one examiner under the supervision and with training from another investigator with over 15 years’ experience with manual head rotation and portable video oculography. It is possible that a less experienced examiner may have greater difficulty achieving a smooth sinusoidal head movement at a consistent velocity, a limitation which may be partially, but not completely, mitigated through use of a metronome.

### 4.2 VVOR and VOR-S properties in healthy controls

A second objective of this study was to determine the influence of head rotation direction and frequency, as well as the effect of age, on VVOR and VOR-S. These effects, once defined, provide a basis for the interpretation of future results from patients suspected of having a cerebellar or vestibular-cerebellar disorder.

The VVOR gain for all frequencies was close to 1.0, which reflects the synergistic interaction of smooth pursuit, optokinetic and VOR responses in maintaining visual fixation across a broad range of head rotation frequencies (7). Similarly, saccades were rarely observed at any frequency. Age also had no significant influence on VVOR gain or rate of saccades, a finding consistent with existing evidence that VOR gains on vHIT remain relatively stable until around 80 years of age (14–16). The small difference in VVOR gain observed between the two directions of rotation, which was greatest at lower frequencies, may be a geometrical consequence of the right-monocular recording technique. This explanation is proposed to account for the slightly higher average gains typical of right-monocular vHIT recordings for head turns to the right, as the right eye has to rotate slightly further to maintain visual fixation (14).

Contrasting with the VVOR, increasing frequency caused VOR-S gain and rates of saccades to increase. Soriano-Reixach et al. (11) also reported an increase in VOR-S gain for manual head rotation from 1.0 to 1.8 Hz. In their study they observed an approximately linear relationship between head rotation frequency and gain. By testing at lower frequencies, we show that the increase in VOR-S gain has a threshold with low gain up to 0.50 Hz, followed by a sharper linear increase as frequency increases beyond 0.50 Hz. This profile aligns with existing theory that the neural substrate for VOR-S is similar to smooth pursuit, which is known to break down over a certain threshold (3). As the efficiency of smooth pursuit declines, additional saccades must be recruited to regain visual fixation. The apparent plateau in saccade rates at approximately 4/s after reaching 0.75 Hz may reflect the physiological limit of how frequently saccades can be generated.

VOR-S gain increased with increasing age, which agrees with evidence describing declining smooth pursuit function with age (17). However, the effect size in our study was small and gains were variable across participants. We found no relationship between age and the rate of VOR-S saccades. Previous studies which have investigated the effect of age on VOR S have produced contradictory results. Studies using rotational chair testing suggest a decline in VOR-S with age (17, 18), while other studies which used manual head rotation reported relatively stable gain below 70 years of age (11). In addition to head rotation frequency, many factors are known to influence VOR-S including participant concentration, alertness, and daily activity levels (11, 19). These factors can increase VOR-S gain and saccade variability, making it difficult to confirm the true effect of age.

### 4.3 Future clinical and research directions

Our study shows the utility of synchronised manual head rotation and video-oculography in testing VVOR and VOR-S. Further reliability could be attained through examiner training to enhance consistency and minimise variability, together with the addition of an improved, live, biofeedback mechanism. A video-oculography software feature that rejects poorly executed VVOR and VOR-S head movements, as well as providing visual or auditory feedback, could help the clinician maintain a consistent sinusoidal velocity profile within a predetermined velocity range. Future software versions which incorporate gain and saccade rate calculation, would further make data analysis more straightforward and time efficient. With such modifications in place, our method could be further validated by comparing results against those obtained using automated rotational chair testing.

This study’s VVOR findings can be expanded upon by exploring the applicability of this method in clinical populations: comparing results in healthy controls with those who have known vestibular, cerebellar, or combined vestibular-cerebellar pathology. The VVOR is most useful in the context of a combined vestibular-cerebellar disorder. However, quantitative data for low-velocity manual head rotation in this population is surprisingly limited. Migliaccio et al. (1), analysed VVOR for low-velocity manual head rotation of up to 63 °/s using scleral search coils. They recorded reduced gain in just four patients with combined vestibular-cerebellar dysfunction compared with a single patient with bilateral vestibulopathy. However, gains overlapped with those of a patient with cerebellar ataxia at low-velocities. As in our study, the investigators used a metronome to pace the oscillations but did not comment on the efficacy of the method. Rey-Martinez et al. (7) reported significantly reduced VVOR gain in five patients with vestibular-cerebellar dysfunction due to CANVAS; however, gain was also reduced in patients with unilateral or bilateral vestibulopathy, probably because of the high rotation velocities that were used. These findings highlight the need for further studies in clinical populations to clarify the optimal frequency and velocity range for identifying combined vestibular-cerebellar dysfunction.

Once the diagnostic capacity of the VVOR is better established, longitudinal studies can assess the potential of this method to monitor changes in function over time, particularly in response to therapeutic interventions.

Although the VOR-S examines similar neural pathways to smooth pursuit, video-oculographic testing of VOR-S during manual head rotation still offers a unique addition to the battery of existing eye movement testing methods by providing an indirect, quantitative assessment. It provides an alternative when equipment required for measuring smooth pursuit is not available or the results are equivocal. Its interpretation is also less affected by saccadic intrusions and other fixation abnormalities (4).

## 5 Conclusion

We have shown that metronome-synchronised video-oculography provides a reliable and valid approach for assessing VVOR and VOR-S in healthy individuals. Its simplicity and cost-effectiveness make it a viable option particularly in settings where access to advanced vestibular testing equipment is limited. The data we provide establish a foundation for future studies. Our results indicate that controlling for age is not necessary for interpretation of VVOR, although practitioners who use a right monocular recording device should be aware of the potential for slightly higher gains for head rotations to the right.

## Data Availability

The datasets presented in this article will be made available on reasonable request. Requests to access the datasets should be directed to rachael.taylor@auckland.ac.nz.

## Supplementary material

SUPPLEMENTARY VIDEO S1 Horizontal VVOR eye movement recording to 0.5 Hz head rotation in a healthy participant. Note the smooth sinusoidal eye movement profile.

SUPPLEMENTARY VIDEO S2 Horizontal VOR-S eye movement recording to 0.5 Hz head rotation in a healthy participant. Note the rapid refixation saccades.

SUPPLEMENTARY VIDEO S3 Horizontal VVOR eye movement recording to 0.5 Hz head rotation in a healthy participant showing a square-wave velocity profile.

SUPPLEMENTARY VIDEO S4 Horizontal VVOR eye movement recording to 0.5 Hz head rotation in a healthy participant with neck resistance. Note the jerky eye movements corresponding to jerky head movement.

SUPPLEMENTARY VIDEO S5 Horizontal VVOR eye movement recording to 0.5 Hz head rotation for the healthy participant in Supplementary Video S4 after reinstruction to try and relax and to not resist the head movement.
