# Supplementary material for: Manual head rotation synchronised to a metronome is a feasible and valid method for assessing visually enhanced vestibulo-ocular reflexes and vestibulo-ocular reflex suppression
Source: Front Neurol. 2026 Apr 22;17:1706773. doi: 10.3389/fneur.2026.1706773 (PMC13143771; doi:10.3389/fneur.2026.1706773)
Supplement: Supplementary file 6 [file Table_1.DOCX]

**Supplementary Table 1**. Summary statistics for intended/expected versus actual measured frequencies and peak velocities of manual head rotation recorded for VVOR and VOR-S. Calculations and measurements are based on a target left and right head turn between 14° and 18.5° either side of the midline (wall markers shown in Figure 1 of the manuscript), corresponding to a total amplitude of exertion between 28° and 37°

Expected peak velocities of head rotation were calculated using the formula:

$$Vpeak=2\pi fA$$

*V*_peak_ is the peak head velocity (deg/s), $f$ is the frequency (Hz), and *A* is the amplitude (degrees) of rotation from the midline to the left or right

| Intended Frequency (Hz) | Mean ± SD Actual Frequency VVOR | Mean ± SD Actual Frequency VOR-S | Expected peak head velocity at ±14° (deg/s) | Expected peak head velocity at ±18.5° (deg/s) | Mean ± SD (deg/s) peak head velocity during VVOR | Mean ± SD (deg/s) peak head velocity during VOR-S |
| --- | --- | --- | --- | --- | --- | --- |
| 0.25 | 0.25 ± 0.01 | 0.25 ± 0.01 | 22.0 | 29.1 | 22.8 ± 3.1 | 20.1 ± 2.4 |
| 0.50 | 0.51 ± 0.02 | 0.52 ± 0.02 | 44.0 | 58.1 | 45.4 ± 5.6 | 44.3 ± 4.1 |
| 0.75 | 0.77 ± 0.02 | 0.78 ± 0.03 | 66.0 | 87.2 | 72.8 ± 8.5 | 73.4 ± 6.5 |
| 1.00 | 1.02 ± 0.01 | 1.03 ± 0.01 | 88.0 | 116.3 | 104.3 ± 13.1 | 107.3 ± 7.3 |
| 1.25 | 1.28 ± 0.03 | 1.28 ± 0.03 | 110.0 | 145.4 | 139.7 ± 18.8 | 141.8 ± 11.0 |
