# Supplementary material for: Manual head rotation synchronised to a metronome is a feasible and valid method for assessing visually enhanced vestibulo-ocular reflexes and vestibulo-ocular reflex suppression
Source: Front Neurol. 2026 Apr 22;17:1706773. doi: 10.3389/fneur.2026.1706773 (PMC13143771; doi:10.3389/fneur.2026.1706773)
Supplement: Supplementary file 7 [file Table_2.docx]

**Supplementary Table 2.** Mean and 95% range (i.e. mean +/- 2SD) for VVOR and VOR-S gain. Data are averaged across the two directions of head movement

| Frequency (Hz) | VVOR gain | | VOR-S gain | |
| --- | --- | --- | --- | --- |
|  | Mean (SD) | Range (Mean +/- 2SD) | Mean (SD) | Range (Mean +/- 2SD) |
| 0.25 | 0.99 (0.06) | 0.86 – 1.11 | 0.15 (0.08) | 0.00 – 0.30 |
| 0.50 | 1.01 (0.06) | 0.88 – 1.13 | 0.20 (0.10) | 0.01 – 0.39 |
| 0.75 | 1.02 (0.06) | 0.90 – 1.13 | 0.30 (0.12) | 0.07 – 0.53 |
| 1.00 | 1.01 (0.06) | 0.89 – 1.13 | 0.40 (0.12) | 0.16 – 0.64 |
| 1.25 | 1.01 (0.06) | 0.89 – 1.13 | 0.51 (0.14) | 0.23 – 0.79 |
