# Supplementary material for: Manual head rotation synchronised to a metronome is a feasible and valid method for assessing visually enhanced vestibulo-ocular reflexes and vestibulo-ocular reflex suppression
Source: Front Neurol. 2026 Apr 22;17:1706773. doi: 10.3389/fneur.2026.1706773 (PMC13143771; doi:10.3389/fneur.2026.1706773)
Supplement: Supplementary file 8 [file Table_3.docx]

**Supplementary Table 3.** Mean and 95% range (i.e. mean +/- 2SD) for VVOR and VOR-S rates of saccades (number per second).

| Frequency (Hz) | VVOR saccades | | VOR-S saccades | |
| --- | --- | --- | --- | --- |
|  | Mean (SD) | Range (Mean +/- 2SD) | Mean (SD) | Range (Mean +/- 2SD) |
| 0.25 | 0.19 (0.22) | 0 – 0.63 | 1.13 (0.78) | 0.00 – 2.69 |
| 0.50 | 0.21 (0.18) | 0 – 0.57 | 2.83 (1.23) | 0.37 – 5.29 |
| 0.75 | 0.24 (0.26) | 0 – 0.76 | 3.74 (1.13) | 1.48 – 6.00 |
| 1.00 | 0.26 (0.21) | 0 – 0.68 | 3.84 (1.04) | 1.76 – 5.92 |
| 1.25 | 0.42 (0.33) | 0 – 1.08 | 3.90 (1.07) | 1.76 – 6.04 |
